# CoreDetector: a flexible and efficient program for core-genome alignment of evolutionary diverse genomes

**DOI:** 10.1093/bioinformatics/btad628

**Published:** 2023-10-25

**Authors:** Mario Fruzangohar, Paula Moolhuijzen, Nicolette Bakaj, Julian Taylor

**Affiliations:** The Biometry Hub, School of Agriculture, Food and Wine, University of Adelaide, Urrbrae, South Australia 5064, Australia; Centre for Crop Disease Management, School of Molecular and Life Sciences, Curtin University, Bentley, Western Australia 6102, Australia; The Biometry Hub, School of Agriculture, Food and Wine, University of Adelaide, Urrbrae, South Australia 5064, Australia; The Biometry Hub, School of Agriculture, Food and Wine, University of Adelaide, Urrbrae, South Australia 5064, Australia

## Abstract

**Motivation:**

Whole genome alignment of eukaryote species remains an important method for the determination of sequence and structural variations and can also be used to ascertain the representative non-redundant core-genome sequence of a population. Many whole genome alignment tools were first developed for the more mature analysis of prokaryote species with few current tools containing the functionality to process larger genomes of eukaryotes as well as genomes of more divergent species. In addition, the functionality of these tools becomes computationally prohibitive due to the significant compute resources needed to handle larger genomes.

**Results:**

In this research, we present CoreDetector, an easy-to-use general-purpose program that can align the core-genome sequences for a range of genome sizes and divergence levels. To illustrate the flexibility of CoreDetector, we conducted alignments of a large set of closely related fungal pathogen and hexaploid wheat cultivar genomes as well as more divergent fly and rodent species genomes. In all cases, compared to existing multiple genome alignment tools, CoreDetector exhibited improved flexibility, efficiency, and competitive accuracy in tested cases.

**Availability and implementation:**

CoreDetector was developed in the cross platform, and easily deployable, Java language. A packaged pipeline is readily executable in a bash terminal without any external need for Perl or Python environments. Installation, example data, and usage instructions for CoreDetector are freely available from https://github.com/mfruzan/CoreDetector.

## 1 Introduction

During evolution of the species, genomes undergo small sequence changes, such as nucleotide substitutions, insertions and deletions, and large-scale duplications, translocations and inversions. More recently, within species, extensive structural variations (SV), such as presence–absence variation, copy number variation, and chromosomal rearrangements, have been identified due to the sequencing and analysis of near complete genomes (Manning *et al.* 2013, Moolhuijzen *et al.* 2018, Moolhuijzen *et al.* 2022, Badet *et al.* 2020, Alouane *et al.* 2021, Bertazzoni *et al.* 2021) Due to advancements in sequencing technologies, such as Oxford Nanopore Technology, PacBio (single molecule long reads), and Illumina (short read), a single species is no longer represented by a single reference genome but by many. Within a sequenced population, the genomic regions found in common to all individuals are referred to as the core genome, and the regions absent in one or more individuals (not present in all individuals) are termed accessory or dispensable regions (Bertazzoni *et al.* 2021). It is the union of the core and accessory genomic regions for a collection of individuals in a species that is referred to as the pangenome, which is larger than the genome of any single individual (Vernikos *et al.* 2015).

To detect and align conserved (homologous) sequences between individuals, multiple sequence alignment (MSA) algorithms are used for smaller genomic regions, represented by protein-coding genes, RNA transcripts, and gene promoter sequences (Van Noorden *et al.* 2014). The aligned homologous regions can then be used in various downstream analyses for motif and protein domain detection, variant discovery, and the construction of phylogenetic trees (Thompson *et al.* 2011). These MSA methods rely on the optimization of an objective function, which is the sum of alignment scores estimated using base substitution matrices (BLOSUM or PAM) and gap penalty scores (Chatzou *et al.* 2016).

The implementation of MSA methods for whole genome alignment (MGA) is inevitably more complicated, and with the rapid development of pangenomes, ranging from small microbes to large plants and vertebrates, fast and efficient MGA algorithms are required to better understand structural changes underlying functional diversity and evolutionary events within and between species. While MSA methods can detect basic evolutionary events, such as nucleotide substitution, insertion and deletion, MGA methods can further reveal larger recombinant events and SVs. The majority of current MGA methods work in two stages. In the first stage, local alignment blocks, called High-scoring Segment Pairs (HSP), are prepared using sequence pairwise alignments, with commonly used tools, such as MUMmer (Marçais *et al.* 2018), LastZ (Harris 2007), GSAlign (Lin and Hsu 2020), MashMap2 (Jain *et al.* 2018), and Minimap2 (Li 2018). In the second stage, post processing, filtering, and merging is conducted to reconcile false-positive HSPs (Armstrong *et al.* 2019). As the local alignment blocks generated in the first stage will miss homologous sequences that diverged further than the sensitivity of the pairwise alignment tool, the post processing of HSPs at a secondary stage or perhaps further alignments becomes essential. Although there are many different approaches to MGA, the progressive alignment method is employed by most state-of-the art tools (Kille *et al.* 2022), such as ProgressiveCactus (Armstrong *et al.* 2020). Optimization of sum of all-against-all alignment scores is a non-deterministic polynomial-time complete problem and heuristic methods are used to solve them (Chatzou *et al.* 2016). The most common heuristic method is progressive alignment, which uses a pre-computed guide-tree (Hogeweg and Hesper 1984). In this method, order of sequence alignment follows the structure of a guide tree where the most closely related sequences are aligned first and the resulting alignment is then aligned to the next closest sequence in the tree. The guide tree itself can be constructed using distance-based Neighbor Joining (NJ) (Saitou and Nei 1987) or Unweighted Pair Group Method with Arithmetic Mean (Murtagh 1984) or character-based maximal parsimony and maximum likelihood methods. Progressive alignment uses a guide phylogeny to divide and order MSA into subsets for merging which, in compute time, scales linearly with the number of sequences and responds poorly for more distant populations (Armstrong *et al.* 2019). Ultimately, tools that provide suitable syntenic sequence outputs are required for identifying relationships between population members as well as understanding their core and accessory genomic elements (Armstrong *et al.* 2019).

The MGA of core and dispensable genomic regions for larger and more genetically diverse individuals in a population can be computationally challenging. A more efficient approach is to perform a core-genome alignment of the conserved sequence to better measure genetic changes between the individuals in the population and show not only the evolutionary relationships within a population but provide further insight into core gene functions and how these may shift over time or geography. Core-genome alignment algorithms ignore the relationships between smaller subsets of genomes, and are therefore inherently more scalable (Treangen *et al.* 2014). Parsnp, e.g. available through the Harvest toolkit (Treangen *et al.* 2014), is a rapid core-genome multi-aligner designed for intraspecific alignments (sequence identity ≥97%) of prokaryote genomes (Treangen *et al.* 2014). Genomes within a specified maximal unique match distance are recruited into the full multiple alignment, which is efficiently stored to a single binary file that encodes the reference genome and a thousand-fold compression of only alignment variants. The final phylogenetic tree is then calculated on these variants (Treangen *et al.* 2014). One unfortunate drawback of these tools is they are usually computationally limited for the analysis of larger and more diverse species.

An alternative computationally expedient approach to capture evolutionary relationships between many genomes rapidly is to use alignment-free methods. Many alignment-free approaches focus on variants of exact matching to convert sequences directly to distances without prior alignment (Haubold *et al.* 2015, Zielezinski *et al.* 2019, Klötzl and Haubold 2020). These approaches, such as Phylonium (Klötzl and Haubold 2020), are extremely fast but there can be technical limits on genome size and the common output generated is a distance matrix, making interrogation of the underlying features in the alignment problematic. Other useful alignment-free approaches include classification machine learning that helps distinguish population members through known targeted features of the sequences (Bakhtiarizadeh *et al.* 2014, Torkzaban *et al.* 2015).

In this research, we created CoreDetector, a simple but fast progressive computational algorithm for core-genome sequence alignment of eukaryote population members with diverse and large genomes. To demonstrate the flexibility of CoreDetector, we compared it to top ranking MGA packages using closely related fungal pathogen and hexaploid wheat genome data as well as more diverse, larger genome datasets from fly and rodent populations. Where possible, we compared the accuracy of the phylogenetic relationships obtained from the resulting core-genome alignments against well-established ground truth phylogenetic trees as well as provide comparative summary information of the core-genome content.

## 2 Materials and methods

### 2.1 CoreDetector implementation

**Figure 1.**
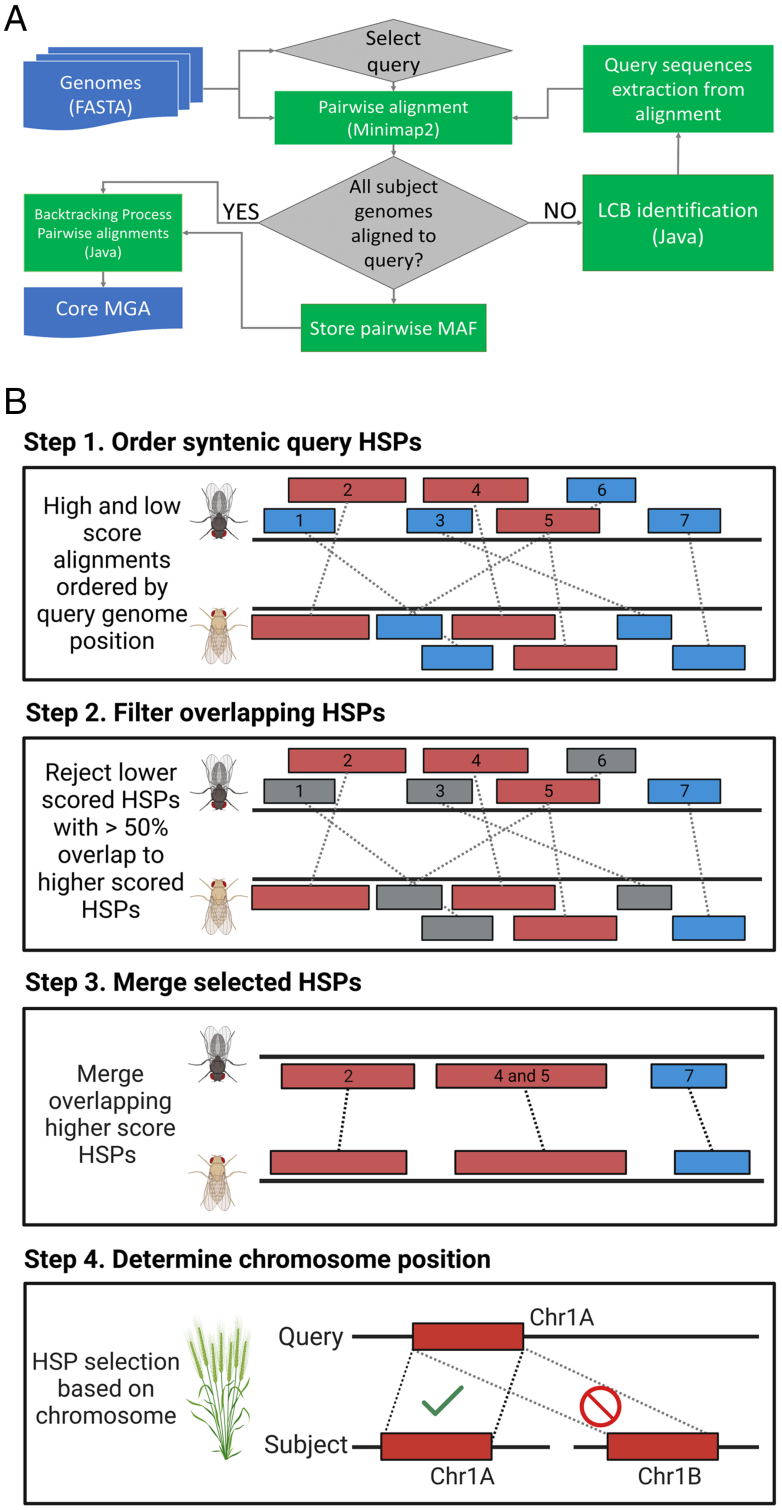
(A) Implementation overview of CoreDetector for the alignment of multiple genomes. (B) The process for LCB/HSP selection. In the query, lower scored HSPs (blue) will be rejected with >50% overlap with a higher scoring HSP (red) (Step 1). Query segments 2, 4, 5, and 7 are selected for further alignment with each dash line representing one HSP (Step 2). HSPs with >1 bp overlap with another similar-scored HSP are merged (Query segments 4 and 5) (Step 3). Query segment selection is determined by subject chromosome information for similar-scored HSPs, where alignments to subject chromosome 1A is selected over chromosome 1B (Step 4).

CoreDetector was designed to identify and generate a multiple core-genome alignment for closely and more distantly related genomes. A single longest genome with the least number of ambiguous bases (non-ATGC) is initially selected as the query from the pool of genomes for pairwise alignment using the fast and efficient pairwise alignment tool, Minimap2 (Li 2018). The query consensus sequence is then extracted from the alignment and then pairwise aligned to the next subject genome and the process repeated until no subject genomes remain (Fig. 1A). Pools of initial local alignments, HSPs, are created in two sensitivity modes to capture high and low divergent homologous sequences (Fig. 1B). The selection of HSPs by CoreDetector is similar to locally co-linear block (LCB) identification and global chaining of co-linear non-overlapping fragments (Abouelhoda and Ohlebusch 2005, Darling *et al.* 2010) but differs in that HSPs are ordered based on ascending position and descending alignment scores, then sequentially added to an ordered list (Fig. 1B, Step 1). A HSP is added to the list if there is no overlap with existing HSPs (HSP 7). In the case of overlap, a new HSP is only added if it has higher score (HSPs 2, 4, and 5 shown in red) than an existing HSP (HSPs 1, 3, and 6 shown in blue), which are then removed from the list (Fig. 1B, Step 2). Overlapping high-scoring HSPs are then merged, HSPs 4 and 5 (Fig. 1B, Step 3). An implied sequence translocation event in the subject, as compared to the query genomes (HSPs 3 and 6), is rejected based on alignment scores. Furthermore, if chromosomal information is available, it is utilized for the identification of true orthologous regions, an especially important feature for polyploidy genomes (Fig. 1B, Step 4). HSPs that are not overlapping, and preferentially have the same chromosomal order (synteny) in both the query and subject, are selected for further alignments (Fig. 1B, Step 3). HSPs shorter than a user defined threshold (default 500 bp) can be excluded from the results to minimize the chance of a false-positive orthologues.

Once all genome alignments are completed, for each HSP entry of the last alignment (MAF file), a backtracking algorithm extracts each subject sequence region from the original pairwise alignment to build a full MSA alignment containing all sequences. The backtracking algorithm then repeatedly updates the sequence alignment profile by adjusting the number and position of gaps in that profile to reconstruct a final core-genome alignment. A more detailed overview of the backtracking algorithm can be found in Supplementary Data S1.

### 2.2 Real datasets used for alignment evaluation

To evaluate the performance of CoreDetector, two real eukaryotic datasets supported by TimeTree (Kumar *et al.* 2017) phylogenetic trees were selected as ground truth for comparison, which included 12 *Drosophila* species (fly), average genome sequence size 180 Mbp (Clark *etal.* 2007), and 34 species of the order Rodentia (rodent), average genome sequence size 2.8 Gbp. The 12 fly genomes were downloaded from the FlyBase (Gramates *et al.* 2022). The 34 rodent genomes were downloaded from the Zoonomia project (Zoonomia 2020). A third published real dataset of 27 fungal *Pyrenophora tritici-repentis* isolates (Moolhuijzen *et al.* 2022) (fungal pathogens), with an average genome sequence size 40 Mbp, were downloaded from the National Centre for Biotechnology Information (NCBI) GenBank. Finally, repeat masked genomes of 10 hexaploid wheat cultivars (*Triticum aestivum*) with average genome size of 14.5 Gbp were downloaded from Ensemble plants database https://ftp.ensemblgenomes.ebi.ac.uk/pub/plants/release-57/fasta/. The fungal pathogen, fly, rodent, and wheat genome accessions and identifiers used in this study are available in Supplementary Data S1.

### 2.3 Alignment software used for comparative run time evaluation

For the fungal pathogen genomes, the run times and memory consumption of software v1.2.3 Mugsy (Angiuoli and Salzberg 2011) and v1.2 Parsnp (Treangen *et al.* 2014) were compared to CoreDetector. For the larger eukaryote genomes, fly, rodent, and hexaploid wheat, additional software packages v2.2.3 ProgressiveCactus (Armstrong *et al.* 2020) and SibeliaZ (Minkin and Medvedev 2020) were also used. The run time options used for each package are available in the Supplementary Data S1. An overview of the alignment methods and features is shown in Table 1.

**Table 1.** Overview of alignment methods for CoreDetector, Mugsy, Parsnp, Progressive Cactus, and SibeliaZ packages to compare run time and memory performance.

Package	Align type	Progressive aligner	Anchor type	Anchor discovery method	Anchor chaining	MSA method
CoreDetector	Core	Yes	Pairwise alignments	Minimap2 (Li 2018)	Recursive merging	Dynamic programming (DP)-based global alignment
Mugsy (Angiuoli and Salzberg 2011)	Whole	Yes	LCBs	Nucmer (Delcher *et al.* 2003)	graph-based chaining	TCoffee (Notredame *et al.* 2000)
Parsnp (Treangen *et al.* 2014)	Core	No	MUMs	Compressed suffix graph	Weighted recursive match chaining	Pecan (Paten *et al.* 2008)
Progressive Cactus (Armstrong *et al.* 2020)	Whole	Yes	Pairwise alignments	LastZ (Harris 2007) and BAR	Cactus alignment filter	libMUSCLE (Edgar 2004)
SibeliaZ (Minkin and Medvedev 2020)	Whole	No	LCBs	Compressed De Buijn graph (Minkin and Medvedev 2020, Raphael *et al.* 2004)	De Bruijn graph	Spoa (Vaser *et al.* 2017)

### 2.4 Computer resources used for alignments

The computer resource consumption was measured on an Oracle cloud computer instance equipped with 16 OCPUS and 128 GB of RAM for the fly dataset and 16 CPUs and 256 GB of RAM for the rodent and hexaploid wheat dataset. ProgressiveCactus was also tested on a larger oracle cloud instance of 32 CPUs and 512 GB of RAM. For the analysis of the wheat fungal pathogens, resource consumption was measured on a Pawsey Nimbus computer cloud instance equipped with 16 VCPUs and 64 GB RAM under the Linux distribution of Ubuntu 20.4.

### 2.5 Phylogenetic tree construction and metrics

For phylogenetic tree evaluation, the resulting output from completed analyses were also compared to the output from two alignment-free packages v1.2 Phylonium (Klötzl and Haubold 2020) and v3.3.0 Skmer (Sarmashghi *et al.* 2019). Phylonium was selected as it was previously used for whole genome phylogenetic analysis of the fungal pathogens (Moolhuijzen *et al.* 2022) and Skmer was tested for the rodent dataset as Phylonium has a 1 Gbp limit on genome size. Table 2 gives an overview of the aligner type, output, and the real genome datasets tested.

**Table 2.** Overview of CoreDetector, Mugsy, Parsnp, ProgressiveCactus, and SibeliaZ and alignment-free Phylonium and Skmer packages and outputs.

Program	Type	Program outputs	Genome data
CoreDetector	Core aligner	MAF; MFA	Fungi, fly, rodent, wheat
Mugsy	MGA aligner	MAF	Fungi, fly, rodent, wheat
Phylonium	Alignment-free	Distance matrix	Fungi, fly
Parsnp (Harvest)	Core aligner	Newick tree and XMFA	Fungi, fly, wheat
ProgressiveCactus	MGA aligner	HAL	Fly, rodent, wheat
Skmer	Alignment-free	Distance matrix	Fly and rodent
SibeliaZ	MGA aligner	MAF	Fly, rodent, and wheat

From alignment MAF files, the fly and rodent phylogenetic trees were generated using a Juke–Cantor genetic distance model with tree construction based on the NJ method available in Geneious prime v2022.1.1 (https://www.geneious.com), and v3.5 PHYLIP software for the fungal pathogens (Retief 2000). A phylogenetic tree was available directly from Parsnp (Treangen *et al.* 2014), and for alignment-free package v1.2 Phylonium (Klötzl and Haubold 2020) and Skmer, trees were constructed directly from the Juke–Cantor-based genetic distance matrix outputs. For ProgressiveCactus, the HAL formatted output files were converted to MAF format using v2.2 hal2maf script (Hickey *et al.* 2013). As ProgressiveCactus and SibeliaZ report both core and accessory alignments, the task to extract the core alignments from the MAF file was integrated into the CoreDetector package pipeline. Ground truth fly and rodent trees, acquired from the TimeTree project (Kumar *et al.* 2017), were then unrooted using R version 4.2.0 package ape v5.6-2 and compared to the phylogenetic fly and rodent NJ trees using TreeCMP (Goluch *et al.* 2020). As there is no ground truth tree available for the fungal pathogens, a matrix comparison of NJ trees was conducted using TreeCMP (Goluch *et al.* 2020). Visual tree comparisons were conducted using R version 4.2.0, packages treeio v1.20.2, dplyr v1.0.10, ggtree v3.4.4, ggplot v2_3.4.0, phangorn v2.11.1, and ape v5.6-2. TreeCMP comparative metrics used included Matching Split, Matching Triplets, Path Difference, Robinson–Foulds metric [RF(0.5)], Quartet, and Unrooted Maximum Agreement Subtree distance (UMAST). The R code and trees generated for the phylogenetic comparative analysis are available from https://github.com/mfruzan/CoreDetector.

### 2.6 Core sequence analysis

The core genome of the fungal pathogens identified by the different packages were searched for low complexity and known transposable element sequences using RepeatMasker (RM) (Chen 2004) v. open-4.0.6 with rmblastn version 2.2.27+ on RepBase (Kohany *et al.* 2006) RM database version 20150807 (taxon = fungi). The nucleotide sequences for the fungal pathogen *Pyrenophora tritici-repentis* isolate M4-predicted genes (Moolhuijzen *et al.* 2018) were searched for using v2.4.0 exonerate (Slater and Birney 2005) (–minintron 10 –maxintron 3000 –model est2genome –percent 90). The core genomes for fly and rodent were searched for gene content using *Drosophila ananassae* (version dana_r1.06_FB2018_04) gene annotations in gene transfer format, downloaded from FlyBase (Gramates *et al.* 2022), and *Mus musculus* (version GCF_000001635.27_GRCm39) gene annotations downloaded from NCBI, using v2.27.1 BedTools intersect (Quinlan 2014) default overlap, respectively.

In addition, gene annotation of hexaploid wheat was downloaded from NCBI and was used to search for gene content in the resulting core-genome alignment. Gene Ontology (GO) slim analysis was conducted on the core gene set using R version 4.2.0 with v2.52.0 biomaRt and v1.58.0 GSEABase packages.

## 3 Results

### 3.1 CoreDetector shows improved speed, memory performance, and flexibility

Software runtime and memory consumption of sequenced-based aligners CoreDetector, v1.2.3 Mugsy (Angiuoli and Salzberg 2011), v1.2 Parsnp (Treangen *et al.* 2014), and v2.2.3 ProgressiveCactus (Armstrong *et al.* 2020) were compared for the selected fungal, fly, rodent, and hexaploid wheat genome datasets (Table 3). In terms of user runtime and memory consumption CoreDetector clearly outperformed the tested alignment software for all four real datasets (fungal pathogens, fly, rodents, and wheat) on the specified resources. Analysis of the closely related 27 fungal pathogen isolates (3.4 GB data size) showed CoreDetector had a faster run time (23.55 min) and lower memory consumption (10 GB) compared to Parsnp (47.31 min and 57 GB). While Mugsy failed to complete, exceeding the maximum resident memory of 64 GB. In the case of the 12 fly species (2.1 GB data size), CoreDetector had a shorter run time and smaller memory consumption, completing successfully in 10 min compared to Progressive cactus and SibeliaZ with runtimes of 35 and 5.5 h, respectively (Table 3). Parsnp failed after 54 min with an error that “aligned regions covered less than 1% of the reference genome.” For the more challenging analysis of the 34 rodent genomes (83 GB data size), CoreDetector completed in 64 min and both Parsnp and Mugsy were unable to complete with “Segmentation fault” and “malloc failed, there is not enough memory” errors, respectively. ProgressiveCactus, which also failed to complete, was then run in a larger cloud instance of 32 CPUs and 512 GB memory and was stopped after 4 weeks as only 20% of data processing had progressed (Table 3). Similarly, for the set of hexaploid wheat genomes (145 GB data size), coreDetector was the only tool that finished under 2 h in an instance of 256 GB memory and 32 CPUs.

**Table 3.** Alignment software user runtime and resident memory performance for the fungal pathogen, fly, rodent, and wheat datasets.^a^

Dataset (data size GB)	Compute resources	Program	User runtime (min)	Max. RAM memory (GB)	Completion status
Fungal pathogen (3.5 GB)	16 CPU; 128 GB RAM	**CoreDetector**	**23.55**	**10**	**Completed**
		Parsnp	47.31	57	Completed
		Mugsy	NA	Out of memory	Failed to complete
Fly (2.1 GB)	16 CPU; 128 GB RAM	**CoreDetector**	**10**	**23**	**Completed**
		Parsnp	NA	NA	Failed to complete
		Progressive Cactus	2100	85	Completed
		SibeliaZ	330	24	Completed
Rodent (87 GB)	32 CPU; 256 GB RAM	**CoreDetector**	**64**	**34**	**Completed**
		Mugsy	NA	Out of memory	Failed to complete
		Parsnp	NA	NA	Failed to complete
		Progressive Cactus	NA	NA	Failed to complete
		SibeliaZ	NA	NA	Failed to complete
Wheat (145 GB)	32 CPU; 256 GB RAM	**CoreDetector**	**118**	**87**	**Completed**
		Mugsy	NA	Out of memory	Failed to complete
		Parsnp	NA	NA	Failed to complete
		Progressive Cactus	NA	NA	Failed to complete
		SibeliaZ	NA	Out of memory	Failed to complete

a Those packages that successfully completed (green) and failed to complete (red) are highlighted. The fastest times and lowest memory consumption for each dataset are in bold.

### 3.2 CoreDetector tree construction is similar to ground truth

The representative “ground truth” phylogenetic trees sourced from the TimeTree project (Kumar *et al.* 2017) for the 12 fly and 34 rodent genomes were then compared to the trees constructed from the MGA and alignment-free packages. The fly and rodent genome accessions, identifiers, and trees used in this study are available in Supplementary Data S1. To measure the dissimilarity of fly ground truth tree compared to the trees generated from the results obtained from CoreDetector, Phylonium, ProgressiveCactus, and SibeliaZ, an array of common distance metrics for unrooted trees were tested (Goluch *et al.* 2020) (Table 4). Across the full range of comparative tree metrics, CoreDetector, ProgressiveCactus, and Phylonium were similar indicating their trees most likely shared similar topology. With the reduced number of Matching Triplets, the tree generated from the results of CoreDetector showed marginally greater similarity to the ground truth compared to trees generated from ProgressiveCactus and Phylonium. Table 4 also indicates the tree generated from the results of SibeliaZ was the most dissimilar to the ground truth tree.

**Table 4.** Phylogenetic tree distance metrics generated from comparing the fly and rodent ground truth tree to trees generated from CoreDetector, Progressive Cactus, SibeliaZ, Phylonium, and Skmer results.

Dataset	Distance metric	Matching triplets	Matching split	Path difference	RF (0.5)	GeoUnrooted	RF weighted (0.5)	Quartet	UMAST
Fly	CoreDetector	4	0	0	0	80.99	131.64	0	0
	Phylonium	10	0	0	0	81.26	132.06	0	0
	ProgressiveCactus	11	0	0	0	80.98	131.63	0	0
	SibeliaZ	43	4	5.65	1	81.17	131.92	21.0	1
Rodent	CoreDetector	471	12	18.43	3	150.17	456.16	672	2
	Skmer	648	20	26.38	5	150.15	456.03	2240	4

In the case of the rodent data, with the exception of CoreDetector, all the alignment tools were unable to analyze the dataset on the specified compute resources used in this study. The assembly-free and alignment-free software Skmer (Sarmashghi *et al.* 2019) provided an alternative for estimating the distances between whole genome skims (low coverage) and allowed us to compare the unrooted phylogenetic trees generated from CoreDetector and Skmer results against the rodent unrooted ground truth phylogenetic tree (Table 4). The numerical comparison of tree metrics indicated that the tree generated from the results of CoreDetector was substantially more similar to the ground truth tree across an array of tree metrics compared to the tree generated from the results of Skmer.

For the fungal pathogen data, as a ground truth tree was not available a TreeCmp (Goluch *et al.* 2020) comparative matrix analysis of the phylogenetic trees generated from CoreDetector, Parsnp, and Phylonium results was conducted. The comparative tree analysis showed that the tree generated from CoreDetector was more similar to the alignment-free Phylonium tree (Table 5).

**Table 5.** The comparison of fungal pathogen NJ phylogenetic trees generated from the results obtained from CoreDetector, Parsnp, and Phylonium.

Distance metric	GeoUnrooted	Matching split	Matching triplets	Path difference	Quartet	RF (0.5)	UMAST
CoreDetector versus Parsnp	0.9874	33	1056	28.14	2846	8	9
CoreDetector versus Phylonium	**0.0005**	**23**	651	24.81	2018	6	**5**
Parsnp versus Phylonium	0.9873	26	**564**	**22.97**	**1508**	**5**	6

Metrics with bold values indicate the topology of the trees generated from the compared software were more closely aligned.

**Figure 2.**
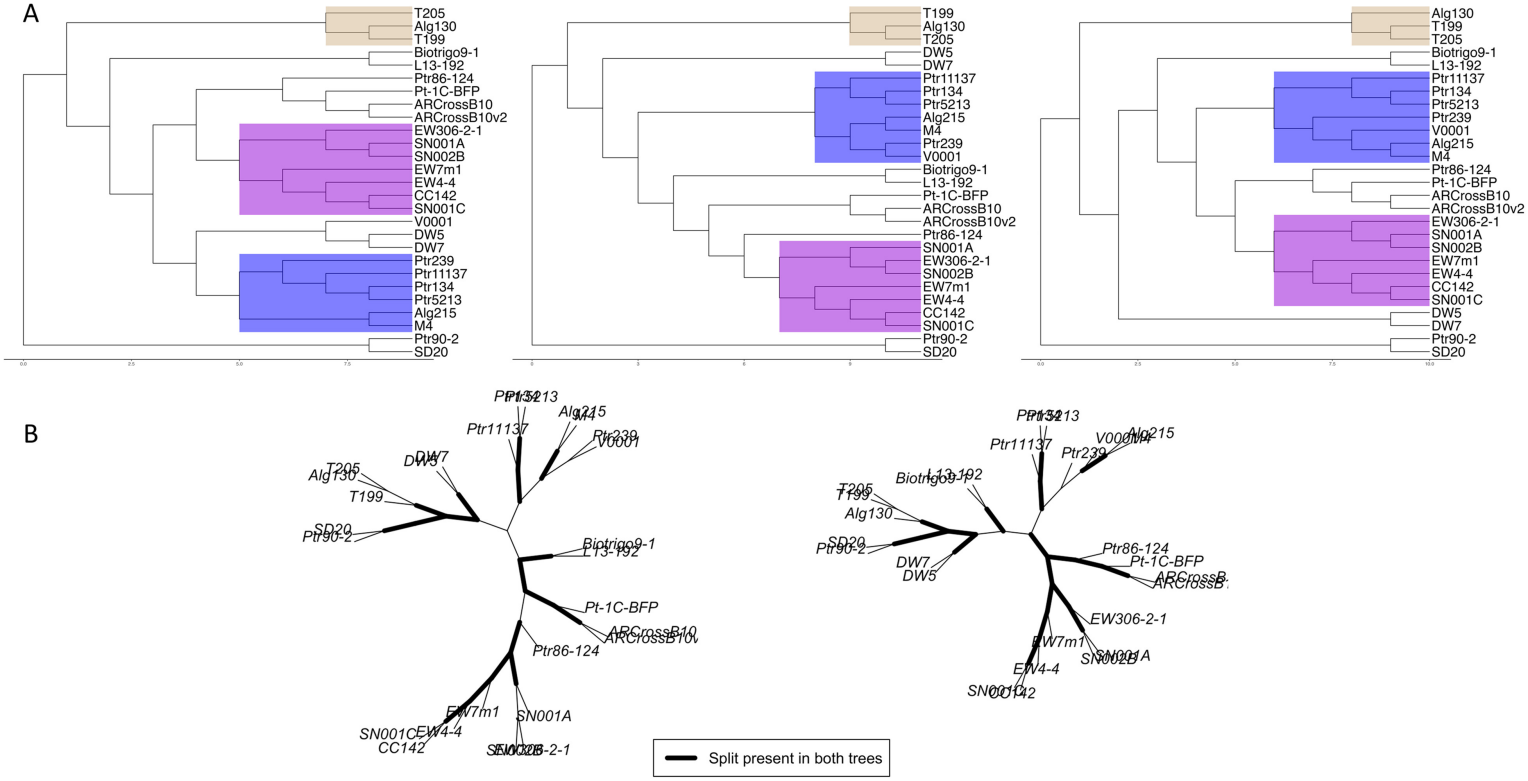
(A) *Pyrenophora tritici-repentis* fungal pathogen phylogenetic tree topology comparisons. Trees generated from CoreDetector (left), Parsnp (center), and Phylonium (right) show three groups related to geographic locations, Europe (violet), Australia (blue), and North Africa (tan). The Ptr isolate identifiers are shown in all three trees. (B) The number of splits in common for Parsnp (left) and Phylonium (right) phylogenetic trees when compared to the CoreDetector phylogenetic tree.

A visual comparison of the fungal pathogen CoreDetector (left), Parsnsp (center), and Phylonium (right) trees showed three cluster groups based on the geographic source of the pathogenic isolates (Fig. 2A) for Europe (violet), Australia (blue), and North Africa (tan) as previously identified (Moolhuijzen *et al.* 2022). Comparison of CoreDetector and Parsnp tree phylogeny found eight clades not in common to each other and 16 splits in common (Fig. 2B, left hand side) and a comparison of CoreDetector and Phylonium tree phylogeny found six clades not in common to each other and 18 splits in common (Fig. 2B, right hand side), again suggesting a closer topology between CoreDetector and the Phylonium generated trees.

### 3.3 Core genome identified by CoreDetector

The core-genome alignments from the programs that ran successfully were then compared for each of the fungal pathogen, fly, and rodent datasets (Table 6). The fungal pathogen CoreDetector and Parsnp core alignment lengths were 31 975 160 and 29 039 970 bp and the number of alignments were 189 486 and 561 736 respectively (Table 6). Compared to the core alignment from Parsnp, the slightly greater CoreDetector core alignment was less fragmented and therefore much more contiguous. The content of the CoreDetector core sequence was searched for protein-coding genes at >90% sequence identity and coverage and 68% of Ptr isolate M4 reference genes (Moolhuijzen *et al.* 2018), 10 599 of 15 458 genes, were represented in the core alignment as compared to 7242 genes for the Parsnp core sequence. For CoreDetector, the gene content represented 41.6% protein-coding gene coverage (13.31 Mbp) of the core alignment length. Furthermore, a total of 3392 core genes were identified by Coredector that were not represented in the Parsnp core alignment. Only 39 440 bp (1.09%) of the core genome was occupied by known retroelements (44 984 bp), DNA transposons (69 805 bp), small RNA (936 bp), simple repeats (175 987 bp), and low complexity regions (25 996 bp).

**Table 6.** Core-genome statistics obtained by different alignment programs for the fungal pathogen, fly, and rodent genome datasets.

Dataset (size)	Program	Fragment number	Core length (Mbp)	Max. length (bp)	N50 length (bp)	Gene coverage (%)^a^	Gene count (%)^b^
Fungal (40 Mb)	CoreDetector	189 486	31.97	51 628	9374	41.6	68.56
	Parsnp	561 736	29.03	28 219	3303	39.3	46.84
Fly (235 Mb)	CoreDetector	679	2.38	28 319	4727	1.93	5.7
	Progressive Cactus	1269	0.08	1000	269	0.06	0.5
	SibeliaZ	2046	0.53	1114	354	0.15	1.1
Rodent (3 Gb)	CoreDetector	5166	14.02	31 220	3882	0.47	6.4
Wheat (14.5 Gb)	CoreDetector	180 597	574	43 913	5191	36.3	56.7

a Percent of gene protein-coding nucleotide sequence that is in the core genome.

b Percent of gene numbers in the core genome.

For the 12 fly species, core alignment lengths for CoreDetector and ProgressiveCactus were 2.38 Mbp and 88.2 kb, respectively (Table 6). Compared to ProgressiveCactus, CoreDetector identified a significantly larger fly core genome (nearly 27-fold greater) and had less fragmented alignments, with approximately half the number of alignments. While for 34 rodent species CoreDetector was the only sequence-based alignment program able to complete (with a 4-week time frame), producing a total core alignment length of 14 Mbp, representing 0.5% of the genome (Table 6). Similarly, for 10 hexaploid wheat genomes, CoreDetector was the only tool to finish and generated a final alignment that covered 36% of the total wheat genic space and more than 50% of the total alignment. The distribution of core genes to each of the wheat genomes A, B, and D were 31.4%, 30.3%, and 35.5%, respectively. The alignment of the homologous actin1 housekeeping genes on chromosomes 4A (TraesCS4A02G264400), 4B (TraesCS4B02G050600), and 4D (TraesCS4D02G050800) were clearly assigned.

**Figure 3.**
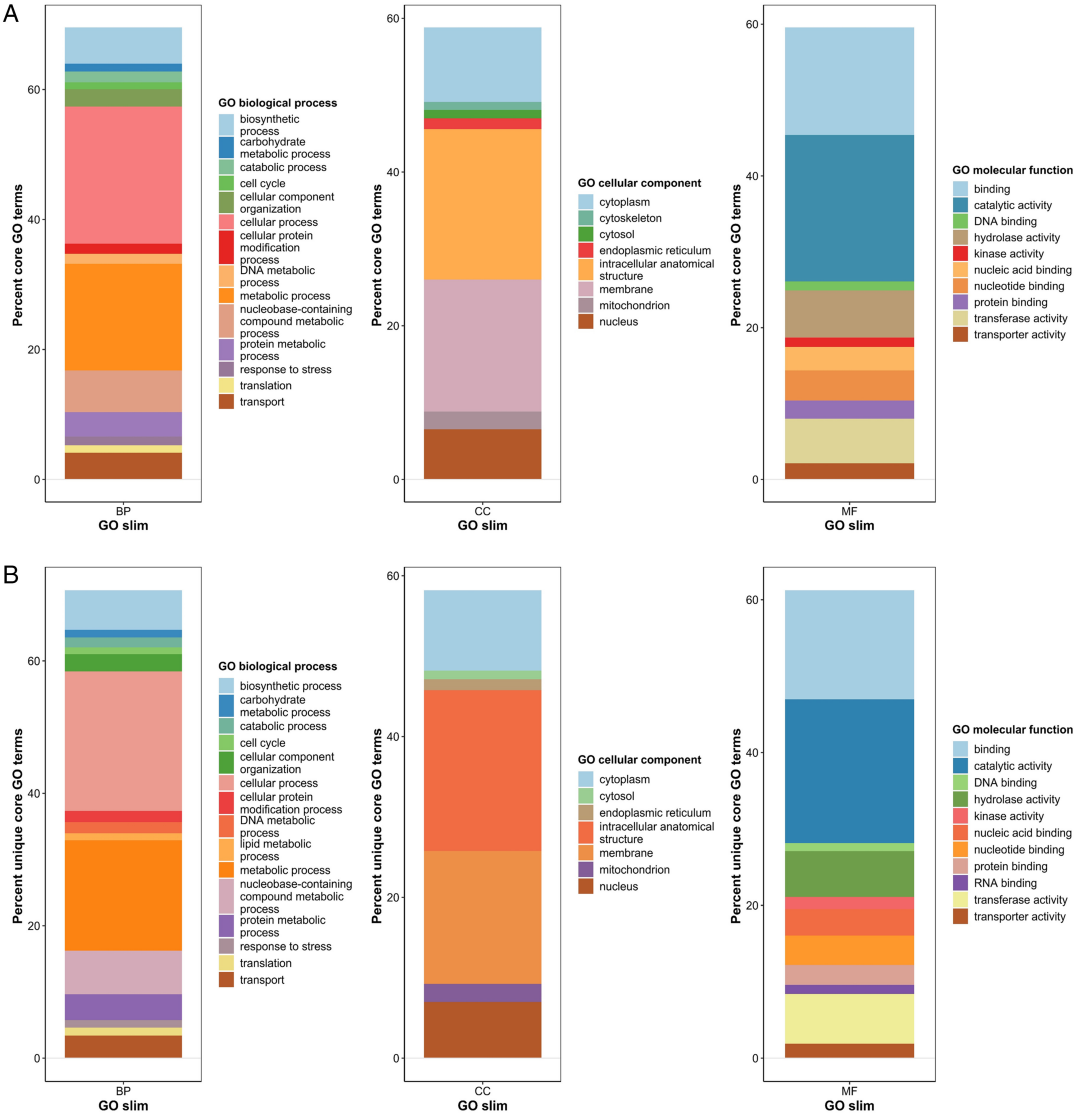
GO slim analysis of fungal pathogen genes identified by CoreDetector (left), cellular components (middle), and molecular functions (right). (A) CoreDetector core genes. (B) Genes identified as core and not identified by other software.

The annotated GOs for the core fungal genes identified by CoreDetector were further investigated to see if any specific groups for molecular functions, biological processes, or cellular components were more abundant in the 3392 uniquely identified genes compared to the core genes (Fig. 3). The overall percentage of genes identified in the core (Fig. 3A) appeared very similar to those uniquely identified (Fig. 3B) and only cytoskeletal cellular components were under-represented and lipid metabolic process overrepresented in the unique core set of genes.

## 4 Discussion

### 4.1 CoreDetector shows improved speed, memory performance, and flexibility

In this study, we have developed CoreDetector, a computational program that is not limited to intraspecific analysis. CoreDetector successfully aligned the core genomes within closely related species, as shown for the fungal pathogen and wheat cultivar genomes used in this study, and also across diverse species, such as the fly (genus) and rodent (order) genome datasets. CoreDetector computationally scaled from the diploid smaller fungal pathogen to larger rodent and hexaploid plant genomes without the need for high-performance computing resources, and in the case of the larger and more diverse rodent dataset, CoreDetector was the only tool that was able to complete in a timely manner in this study.

### 4.2 CoreDetector implements novel features

An important feature of CoreDetector is its independence from any guide tree, which can be extremely useful for multi-genome projects on new populations and non-model organisms, where a single reference genome is unknown. CoreDetector also provides an option to use more efficient pairwise alignment tools with two modes of sensitivity that improves the capture of homologous sequences from divergent populations and minimizes false positives.

The additional ability of CoreDetector to use available chromosome information to aid the identification of true orthologues is a feature we believe is implemented for the first time in this study. For hexaploid wheat, individual cultivars have three near duplicated diploid genomes A, B, and D (Appels *et al.* 2018) and so the use of chromosome information in the wheat genome alignment became an especially important feature that allowed us to more accurately identify the correct orthologous sequences.

### 4.3 CoreDetector outperforms existing alignment software

CoreDetector, outperformed the only other core-genome aligner software package compared, Parsnp, in both runtime and memory use on the specified compute resources. It would be unreasonable to expect Parsnp to be able the process the larger more diverse genomes as it was designed for intraspecific smaller prokaryote genome alignment (Treangen *et al.* 2014). However, other MGA tools also struggled, with most exceeding the maximum memory allowable in this study. CoreDetector was the only software package capable of processing all three datasets using the specified resources. This highlights a definitive niche for CoreDetector. The computational success of CoreDetector is in part due to the fast anchor discovery method provided by Minimap2 (Li 2018) that is dramatically more efficient than the tools, such as Nucmer (Notredame *et al.* 2000) and LastZ (Harris 2007), that are used as pairwise aligners in Mugsy and ProgressiveCactus, respectively. CoreDetector also contains a modern DP-based global alignment algorithm that is also likely to be more efficient than older MSA methods, such as TCoffee (Notredame *et al.* 2000), Pecan (Vaser *et al.* 2017), and MUSCLE (Edgar 2004), implemented in the comparative MGA tools used in this research. Powerhouse MGA software tools SibeliaZ (Minkin and Medvedev 2020) and ProgressiveCactus were both developed for the alignment of larger genomes, and while SibeliaZ was shown previously to outperform ProgressiveCactus on a single machine in speed, ProgressiveCactus was recommended as the better option for more divergent genomes (Treangen *et al.* 2014). Given the inability of ProgressiveCactus to complete the alignment of the rodent dataset, CoreDetector becomes well positioned as a faster and more flexible option to enable such studies.

### 4.4 Core-genome alignment phylogenetic trees are closer to ground truth

The assessment of the validity of alignment outputs becomes difficult without a known correct outcome or ground truth. In this study, we chose to use two datasets, fly and rodent, where ground truth phylogenetic trees had been highly curated from published peer-reviewed studies, made available through the TimeTree project (Kumar *et al.* 2017). When compared to the fly ground truth phylogenetic tree, CoreDetector showed improved metrics when compared to the tree generated from the results of one of the leading alignment-free software tools available, Phylonium (Klötzl and Haubold 2020). The difference of competitive accuracy from Phylonium may be due to its more common use with closely related multiple genomes where it provides startling good performance metrics (Zielezinski *et al.* 2019). For the rodent data, CoreDetector tree could only be compared to Skmer with CoreDetector showing comparable similarity to the ground truth tree. For the fungal pathogens the correct clustering of isolates in the phylogenetic tree produced from the results of CoreDetector is supported by the geography of the collected isolates, as previously found (Moolhuijzen *et al.* 2022).

### 4.5 Core-genome sequence identified by CoreDetector reflect a range of evolutionary divergence

It is well known that protein-coding gene sequences are more conserved than intergenic regions within and across different species. In this study, the 12 Drosophila species dataset had quite variable genomic sizes, from *Drosophila simulans* at 124.9 Mb to *Drosophila willistoni* at 235.5 Mb (Clark *etal.* 2007, Hjelmen *et al.* 2019) and protein-coding sequences that ranged from 38.9 Mb in *Drosophila melanogaster* to 65.4 Mb in *D.willistoni*. It is therefore possible that the final core alignment length (2.38 Mb) obtained by CoreDetector, which represented 6.1% of the smallest protein-coding sequence, does reflect a significant level of divergence for a set of species, which diverged nearly 40 million years ago (Kumar *et al.* 2017). Furthermore, despite the low retrieval of core sequence the rodent dataset, it is probable that this too reflects the much earlier divergence time for the rodent order over 52 million years ago (Kumar *et al.* 2017).

Interestingly, for the fungal pathogen isolates the core-genome predicted protein content (68%) was larger than a previous orthologous protein clustering analysis of predicted genes, which was 56% based on clustering of orthologous proteins (Moolhuijzen *et al.* 2022). As the fungal pathogen genomes were represented by a range of sequencing technologies (from fragmented Illumina to chromosome level PacBio sequenced assemblies) and given the core sequence identified is dependent on genome completeness, the identification a larger core sequence by CoreDetector is impressive and provides additional genomic sequence to help gain further insight into core genomes of different populations.

## Supplementary Material

btad628_Supplementary_Data

## Data Availability

All data generated or analyzed during this study are included and can be accessed in this published article (and Supplementary Data S1). CoreDetector, a step-by-step manual and supporting scripts used in this study are freely available from GitHub at https://github.com/mfruzan/CoreDetector.

## Code availability

Our tool is open source and freely available at https://github.com/mfruzan/CoreDetector.
