# Supplementary material for: CoreDetector: a flexible and efficient program for core-genome alignment of evolutionary diverse genomes
Source: Bioinformatics. 2023 Oct 25;39(11):btad628. doi: 10.1093/bioinformatics/btad628 (PMC10663985; doi:10.1093/bioinformatics/btad628)
Supplement: btad628_Supplementary_Data [file btad628_supplementary_data.zip › CoreDetector_Supplementary_Data_Revised.docx]

# CoreDetector: A flexible and efficient program for core-genome alignment of evolutionary diverse genomes

Mario Fruzangohar1*, Paula Moolhuijzen2, Nicolette Bakaj1, Julian Taylor1

1The Biometry Hub, School of Agriculture, Food and Wine, University of Adelaide, Australia

2Centre for Crop and Disease Management, School of Molecular and Life Sciences, Curtin University, Bentley, WA 6102, Australia

*Corresponding author

Table of Contents

[CoreDetector: A flexible and efficient program for core-genome alignment of evolutionary diverse genomes 1](#_Toc145959839)

[Supplementary Methods Notes 2](#_Toc145959840)

[Extraction of query consensus sequence from pairwise alignment (MAF) 2](#_Toc145959841)

[CoreDetector Backtracking algorithm 3](#_Toc145959842)

[Complexity of Backtracking algorithm 4](#_Toc145959843)

[Selecting homologous sequences 4](#_Toc145959844)

[Experiment 1) Analysis of 27 fungal pathogen (Ptr) genomes 5](#_Toc145959845)

[Table S1.*Pyrenophora tritici-repentis* (Ptr) isolate genomes 5](#_Toc145959846)

[Commands for fungal pathogen analysis 6](#_Toc145959847)

[Experiment 2) Analysis of 12 Drosophila genomes 6](#_Toc145959848)

[Table S2 Drosophila genus genomes 6](#_Toc145959849)

[Commands for fly analysis 6](#_Toc145959850)

[Experiment 3) Analysis of 34 Rodent genomes 8](#_Toc145959851)

[Table S3 Rodent order genomes 8](#_Toc145959852)

[Commands for rodent analysis 9](#_Toc145959853)

[Experiment 4) Analysis of 10 wheat cultivar genomes 9](#_Toc145959854)

[Table S4 Wheat cultivar genomes 10](#_Toc145959855)

[Commands for wheat analysis 10](#_Toc145959856)

# Supplementary Methods Notes

## Extraction of query consensus sequence from pairwise alignment (MAF)

For each pairwise genome alignment, post locally co-linear block (LCB) identification and block chaining of co-linear overlapping fragments; for each entry in the pair-wise MAF file, one ungapped FASTA contig is generated using CoreDetector *maf2fasta* java task option (Figure S1). Sequence indices, constructed from the query contig name, start and end positions, are used in the final backtracking algorithm to identify the aligned subject sequence:

The query FASTA sequence generated is then used to align against the next subject genome.

**
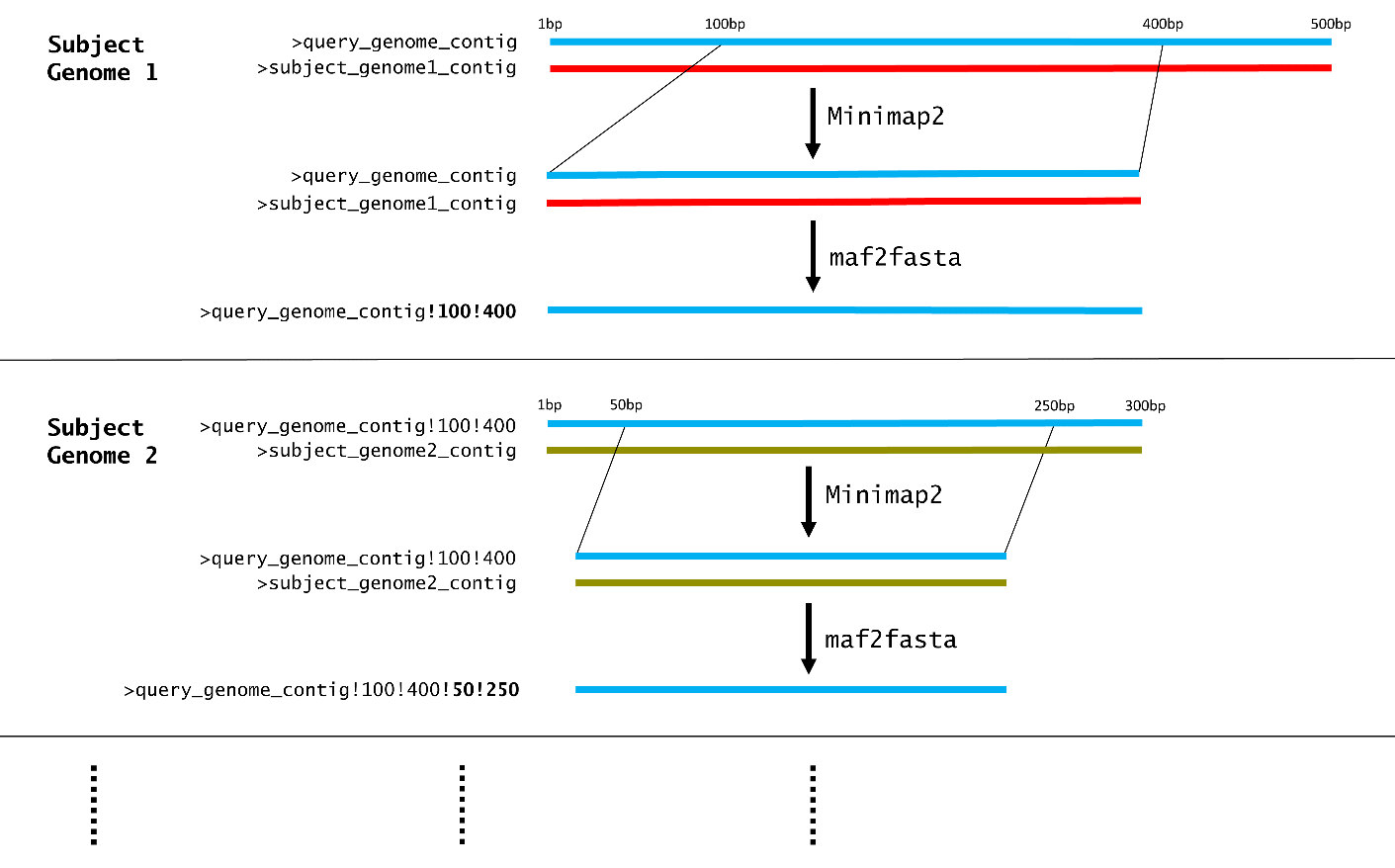
**

**Fig S1**: *Iterative pairwise alignment and query extraction process: Query genome is pairwise aligned to subject genome 1; maf2fasta extracts and construct the new query genome contig that is pairwise aligned to the subject genome 2; this process is repeated until no subject genomes are left.*

## CoreDetector Backtracking algorithm

Once all genome pairwise alignments are completed, all *N - 1* generated pair-wise MAF files are processed recursively (starting from the last MAF file through to the first one). For each HSP entry of the last MAF file, a backtracking algorithm extracts each subject sequence part (based on the previously constructed query indices) and finally builds an MSA entry containing *N* sequences. MSA entries are written as a final output MAF file or all concatenated into a final output FASTA file containing *N* contigs.

Given *N* genomes results in *N* *- 1* pairwise MAF file alignments, each pairwise alignment is accessed through an array defined as *MAF[1 .. N - 1]*. Foreach pairwise MAF alignment, let *msaK* be a list variable of *K* sequences that represent the multiple sequence alignment profile of the *K* sequences, and let the function *getHomolog (hsp.query, maf)* be a function that searches for the query part of the input *hsp* in the input pairwise alignment *maf* file and returns an *hsp* subject.

The adjustment of alignment gaps occurs using an algorithm that consists of two processes. First process is *gap surveyor* that essentially prepares a map of gap positions and their numbers. The second process is *gap merger* that merges the gap survey of 2 sequences. The algorithm starts with preparing a gap survey for the query sequence as a Java Map object. When a subject profile is added to the end of alignment profile of *msaK*, the gaps survey is merged with each previous sequence’s gap survey profile in *msaK* and as a result of merging, all sequences in the profile and their gap surveys are updated.

## Complexity of Backtracking algorithm

Order of the algorithm is a function of number of genomes (N), level of divergence between genomes (D) and length of genomes (L). The first loop in the algorithm has an order equal to number of HSPs in the final pair-wise alignment, that itself is a function of N, L and D and can be shown with . Then the order of the algorithm is .

## Selecting homologous sequences

The pairwise aligner is run in low and high sensitivity modes and the results of the two runs are merged into a single MAF file. In low sensitivity mode, more similar sequences are captured and in high sensitivity mode more divergent sequences are detected. Adjacent hits from the two runs are then merged into larger hits.

As pairwise alignments can report multiple hits (HSPs) due to paralogue sequences (duplication) or by chance, CoreDetector can use the chromosome number (if available) and relative position of an HSP in that chromosome to select the best hit that likely reflects the true orthologs. HSPs shorter than a user selected threshold (default 500bp) is excluded from the results to minimize the chance of a false positive orthologs.

# Experiment 1) Analysis of 27 fungal pathogen (Ptr) genomes

## Table S1.*Pyrenophora tritici-repentis* (Ptr) isolate genomes

| Assembly Accession | Strain | Sequencing techonolgy | Length | Assembly Level |
| --- | --- | --- | --- | --- |
| GCA_003171515.3 | M4 | PacBio | 40738834 | Chromosome |
| GCA_003171545.1 | ARCrossB10v1 | Illumina | 33619417 | Scaffold |
| GCA_003231325.2 | Ptr134 | Illumina | 40622625 | Chromosome |
| GCA_003231345.1 | Ptr5213 | Illumina | 34183844 | Scaffold |
| GCA_003231355.1 | Ptr11137 | Illumina | 33921061 | Scaffold |
| GCA_003231365.1 | Ptr239 | Illumina | 34483177 | Scaffold |
| GCA_003231415.2 | DW5 | PacBio | 40616955 | Chromosome |
| GCA_003231425.2 | 86-124 | PacBio | 40927694 | Chromosome |
| GCA_008692205.1 | V0001 | PacBio | 40136298 | Contig |
| GCA_018492725.1 | AR CrossB10v2 | PacBio | 39876747 | Contig |
| GCA_022544795.1 | Alg130 | Illumina | 34824145 | Scaffold |
| GCA_022544805.1 | Alg215 | Illumina | 34570617 | Scaffold |
| GCA_022578365.1 | T205 | Illumina | 34133756 | Scaffold |
| GCA_022578395.1 | T199 | Illumina | 34281657 | Scaffold |
| GCA_022788405.1 | EW4-4 | Illumina | 34368509 | Scaffold |
| GCA_022788415.1 | SN001A | Illumina | 34152043 | Scaffold |
| GCA_022788425.1 | SN002B | Illumina | 35159469 | Scaffold |
| GCA_022788435.1 | EW7m1 | Illumina | 34228317 | Scaffold |
| GCA_022788445.1 | SN001C | Illumina | 34292940 | Scaffold |
| GCA_022788505.1 | EW306-2-1 | Illumina | 34540381 | Scaffold |
| GCA_022788515.1 | CC142 | Illumina | 34345883 | Scaffold |
| GCA_022813025.1 | Biotrigo9-1 | PacBio | 42003943 | Chromosome |
| GCA_022813065.1 | L13-192 | PacBio | 36964309 | Chromosome |
| GCA_022837075.1 | Ptr90-2 | Pacbio | 39319516 | Chromosome |
| GCA_000149985.1 | Pt-1C-BFP | Sanger | 37840464 | Scaffold |
|  | *DW7 | Illumina |  | Scaffold |
|  | *SD20 | Illumina |  | Scaffold |

*Assembly originally sourced from DOI: 10.1186/s12864-018-4680-3 published in 2018.

## Commands for fungal pathogen analysis

CoreDetector:

*/usr/bin/time -v "pipeline_Minimap.sh -g ptr_genomes.txt -o coredector_ptr27 -d 10 -n 16"*

Mugsy:

Parsnp:

Phylonium:

# Experiment 2) Analysis of 12 Drosophila genomes

## Table S2 Drosophila genus genomes

| **Assembly Accession** | **Organism Name** | **Length** | **Assembly Level** |
| --- | --- | --- | --- |
| GCA_000001215.4 | Drosophila melanogaster | 143706478 | Chromosome |
| GCA_003285735.2 | Drosophila virilis | 189443829 | Contig |
| GCA_003286085.2 | Drosophila persimilis | 195512972 | Contig |
| GCA_003286155.2 | Drosophila erecta | 146538397 | Contig |
| GCA_004382195.2 | Drosophila sechellia | 153084571 | Chromosome |
| GCA_009870125.2 | Drosophila pseudoobscura | 163266851 | Chromosome |
| GCA_016746365.2 | Drosophila yakuba | 147883098 | Chromosome |
| GCA_016746395.2 | Drosophila simulans | 131663590 | Chromosome |
| GCA_017639315.2 | Drosophila ananassae | 213817545 | Chromosome |
| GCA_018153295.1 | Drosophila grimshawi | 191382978 | Contig |
| GCA_018153725.1 | Drosophila mojavensis | 163170721 | Contig |
| GCA_018902025.2 | Drosophila willistoni | 246985538 | Chromosome |

## Commands for fly analysis

CoreDetector:

*./pipeline_Minimap.sh -g /path/to/genomes.txt -o /output/folder -d 40 -n 32*

Parsnp:

Phylonium:

1. Run Phylonium

*time ./phylonium mel.fasta sec.fasta sim.fasta yak.fasta ere.fasta*

*ana.fasta pse.fasta per.fasta wil.fasta vir.fasta moj.fasta gri.fasta > phylip.mat*

1. Install mattools from https://github.com/EvolBioInf/mattools

ProgressiveCactus:

1. Make sure you update TOIL directory to a directory that has enough space:
2. Run halStats to generate phylogenetics tree from hal file:
3. Convert hal to maf format:
4. Analysis of maf file and the extraction of the core sequence:

SibeliaZ:

Using 32 cores and 256GB of RAM

1. Run Sibeliaz
2. Analysis of maf file and the extraction of the core sequence:

# Experiment 3) Analysis of 34 Rodent genomes

## Table S3 Rodent order genomes

| Assembly Accession | Organism Name | Length | Assembly Level |
| --- | --- | --- | --- |
| GCA_004027535.1 | Acomys cahirinus | 2306070819 | Scaffold |
| GCA_004027875.1 | Aplodontia rufa | 3005535537 | Scaffold |
| GCA_001984765.1 | Castor canadensis | 2518306565 | Scaffold |
| GCA_004027575.1 | Cricetomys gambianus | 2397721602 | Scaffold |
| GCA_000223135.1 | Cricetulus griseus | 2399770464 | Scaffold |
| GCA_000151885.2 | Dipodomys ordii | 2236368823 | Scaffold |
| GCA_004024685.1 | Dipodomys stephensi | 2346418196 | Scaffold |
| GCA_001685075.1 | Ellobius lutescens | 2353188398 | Scaffold |
| GCA_001685095.1 | Ellobius talpinus | 2265966243 | Scaffold |
| GCA_004027185.1 | Glis glis | 2462087207 | Scaffold |
| GCA_004027655.1 | Graphiurus murinus | 2815014629 | Scaffold |
| GCA_016881025.1 | Ictidomys tridecemlineatus | 2478949113 | Chromosome |
| GCA_020740685.1 | Jaculus jaculus | 2863848715 | Chromosome |
| GCA_001458135.2 | Marmota marmota marmota | 2506852125 | Scaffold |
| GCA_002204375.1 | Meriones unguiculatus | 2523107715 | Scaffold |
| GCA_017639785.1 | Mesocricetus auratus | 2457062007 | Scaffold |
| GCA_000317375.1 | Microtus ochrogaster | 2287340943 | Chromosome |
| GCA_900094665.2 | Mus caroli | 2553112587 | Chromosome |
| GCA_000001635.9 | Mus musculus | 2728206152 | Chromosome |
| GCA_900095145.2 | Mus pahari | 2475012951 | Chromosome |
| GCA_921997135.2 | Mus spretus | 2546527799 | Chromosome |
| GCA_004027005.1 | Muscardinus avellanarius | 2527147110 | Scaffold |
| GCA_000622305.1 | Nannospalax galili | 3061408210 | Scaffold |
| GCA_004026605.1 | Ondatra zibethicus | 2562752769 | Scaffold |
| GCA_903995425.1 | Onychomys torridus | 2468394440 | Chromosome |
| *GCA_004027895.1 | Orientallactaga bullata | 3093575781 | Scaffold |
| GCA_023159225.1 | Perognathus longimembris pacificus | 2212099196 | Chromosome |
| GCA_003704035.3 | Peromyscus maniculatus bairdii | 2512423440 | Chromosome |
| GCA_907164565.1 | Psammomys obesus | 2364980841 | Scaffold |
| GCA_015227675.2 | Rattus norvegicus | 2647899415 | Chromosome |
| GCA_004025045.1 | Sigmodon hispidus | 2730600022 | Scaffold |
| GCA_002406435.1 | Spermophilus dauricus | 3106271744 | Scaffold |
| GCA_004024805.1 | Xerus inauris | 2601418404 | Scaffold |
| GCA_004024765.1 | Zapus hudsonius | 2611189839 | Scaffold |

- Note Orientallactaga bullata (Gobi jerboa)= Allactaga_bullata

## Commands for rodent analysis

CoreDetector:

*time ./pipeline.sh -g 34genomes_noindex.txt -o Minimap_2 -d 40 -n 32*

Parsnp (Harvest):

Resources: We allocated a machine with 16 OCPU and 256GB RAM.

Mugsy:

Allocated machine with 16 OCPU and 256GB RAM.

*time ./mugsy -directory /path/to/output --prefix Rodentgenomes*

*comys_cahirinus.fna Allactaga_bullata.fna Aplodontia_rufa.fna*

*Castor_canadensis.fna Cricetomys_gambianus.fna Cricetulus_griseus.fna*

*Dipodomys_ordii.fna Dipodomys_stephensi.fna Ellobius_lutescens_v1.fna*

*Ellobius_talpinus.fna GliGli.fna Graphiurus_murinus.fna*

*Ictidomys_tridecemlineatus.fna Jaculus_jaculus.fna Marmota_marmota.fna*

*Meriones_unguiculatus.fna Mesocricetus_auratus.fna Microtus_ochrogaster.fna*

*Mus_caroli.fna Mus_pahari.fna Mus_spretus.fna Muscardinus_avellanarius_v1.fna*

*Nannospalax_galili.fna Ondatra_zibethicus.fna Onychomys_torridus.fna*

*Perognathus_longimembris.fna Peromyscus_maniculatus.fna Psammomys_obesus.fna*

*Rattus_norvegicus.fna Sigmodon_hispidus.fna Spermophilus_dauricus.fna*

*Xerus_inauris.fna Zapus_hudsonius.fna mus_musculus_v39.fna*

ProgressiveCactus:

Allocated machine with 32 OCPU and 512GB RAM.

1. Make sure you update TOIL directory to a directory that has enough space:
2. Run ProgressiveCactus

Skmer:

Allocated machine with 32 Cores and 128GB of RAM

# Experiment 4) Analysis of 10 wheat cultivar genomes

## Table S4 Wheat cultivar genomes

| Assembly Accession | cultivar name | Length | Assembly Level |
| --- | --- | --- | --- |
| GCA_018294505.1 | Chinese Spring | 14.6 Gb | Chromosome |
| GCA_903993795.1 | Jagger | 14.5 Gb | Chromosome |
| GCA_903994195.1 | Julius | 14.5 Gb | Chromosome |
| GCA_903993975.1 | Lancer | 14.3 Gb | Chromosome |
| GCA_903994175.1 | Mace | 14.4 Gb | Chromosome |
| GCA_903994185.1 | SY-Mattis | 14.9 Gb | Chromosome |
| GCA_903993985.1 | ArinaLr | 14.6 Gb | Chromosome |
| GCA_904066035.1 | Norin61 | 14.9 Gb | Chromosome |
| GCA_903995565.1 | Landmark | 14.4 Gb | Chromosome |
| GCA_903994155.1 | Stanley | 14.5 Gb | Chromosome |

## Commands for wheat analysis

Allocated machine with 32 OCPU and 256GB RAM.

CoreDetector:

*time ./pipeline.sh -g 10genomes.txt -o Minimap -d 5 -n 32 -c 1*

parameter *‘-c 1’* will enable chromosome matching, because all 10 wheat genome assemblies are at chromosome level and their contig names start with chromosome number.

Mugsy:

*time mugsy --directory /path/to/output --prefix wheatgenomes CS_rm.fa arina_rm.fa jagger_rm.fa julius_rm.fa lancer_rm.fa landmark_rm.fa mace_rm.fa mattis_rm.fa norin61_rm.fa stanley_rm.fa*

ProgressiveCactus:

*time cactus /path/to/cactus/output /path/to/genomes/10genomes.txt wheat_msa.hal*

Parsnp (Harvest):

SibeliaZ:

*time sibeliaz -t 32 CS2.1/CS_rm.fa SY-Mattis/mattis_rm.fa arinaLr/arina_rm.fa jagger/jagger_rm.fa julius/julius_rm.fa lancer/lancer_rm.fa landmark/landmark_rm.fa mace/mace_rm.fa norin61/norin61_rm.fa stanley/stanley_rm.fa*
