# Supplementary figures and images for: CoreDetector: a flexible and efficient program for core-genome alignment of evolutionary diverse genomes
Source: Bioinformatics. 2023 Oct 25;39(11):btad628. doi: 10.1093/bioinformatics/btad628 (PMC10663985; doi:10.1093/bioinformatics/btad628)

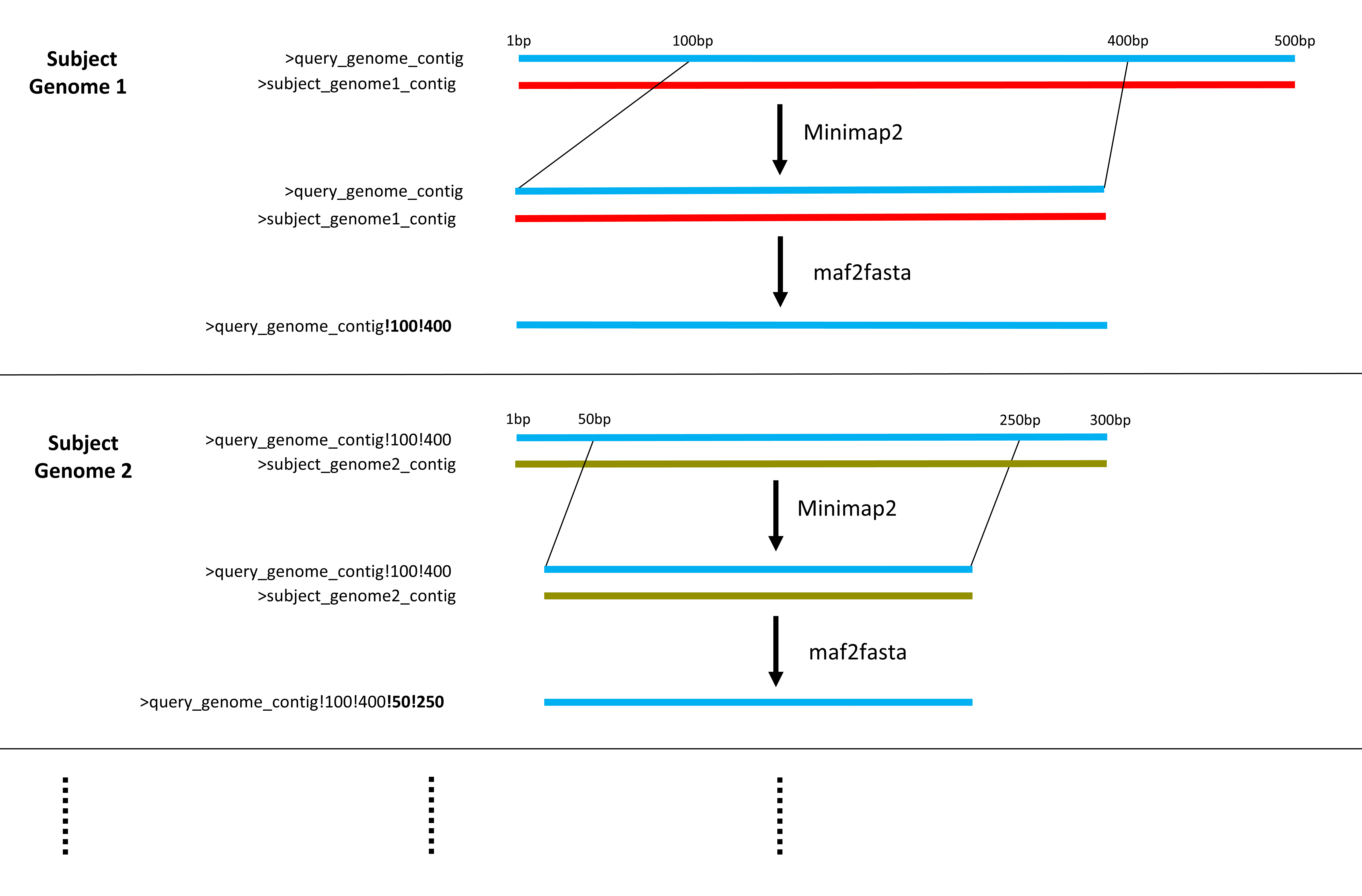

Supplement: btad628_Supplementary_Data [file btad628_supplementary_data.zip › Figure-S1.png]
